# Measurement of the Pitch Diameter of Buttress Threads Using a Touch-Trigger Probe on a CNC Machine Tool

**DOI:** 10.3390/ma19153293

**Published:** 2026-08-03

**Authors:** Bartłomiej Krawczyk, Piotr Szablewski, Sylwia Wencel

**Affiliations:** 1 Pratt & Whitney Kalisz, Elektryczna 4a, 62-800 Kalisz, Poland; piotr.szablewski@prattwhitney.com; 2 Institute of Mechanical Engineering, University of Kalisz, Wojciecha Bogusławskiego 2, 62-800 Kalisz, Poland; 3 Faculty of Production Engineering and Materials Technology, Czestochowa University of Technology, Av. Armii Krajowej 19, 42-201 Czestochowa, Poland

**Keywords:** touch-trigger probe, CNC, thread, pitch diameter, measurement

## Abstract

This research evaluates the accuracy and repeatability of a novel procedure for measuring pitch diameter using a touch-trigger probe in a production environment. Experiments were performed on a WFL M40 machine equipped with a Renishaw RMP600 strain gauge probe, using different thread types and machine tools. The results were validated by comparison with the traditional three-wire method, which is regarded as a high-precision reference for pitch diameter measurement. Statistical analyses, including the Shapiro–Wilk test, ANOVA, and Tukey HSD post hoc tests, were applied to assess error distribution and differences between thread types and machines. The proposed method demonstrated satisfactory accuracy and repeatability, with an error spread of approximately ±0.015 mm, achieving measurement results within the manufacturing tolerance range (±0.07 mm). The study also showed that measurement accuracy is influenced by factors such as thread angle and *Z*-axis positioning. Overall, the method proved to be reliable for production use, offering a good balance between measurement accuracy and operational efficiency, although minor adjustments may be needed for automatic correction routines.

## 1. Introduction

The concept of Industry 4.0 integrates cyber–physical systems, the Internet of Things (IoT), Cloud Computing, and Artificial Intelligence (AI) to enable data-driven manufacturing and smart factories. This transformation also extends to measurement processes, giving rise to concepts such as In-Process Metrology [1], On-Machine Measurement [2,3], and Cyber–Physical Manufacturing Metrology [4]. These approaches integrate measurement directly into manufacturing processes, enabling continuous process monitoring and automatic data analysis. As a result, process deviations can be detected and corrected before they lead to significant quality issues. The challenges associated with Metrology 4.0 have been discussed extensively in the literature [5,6,7]. Researchers emphasize the importance of ensuring the repeatability, reliability, and stability of automated measurements, particularly under shop-floor conditions. Evaluating the measurement uncertainty arising from the combined influence of machine tool errors and measuring system errors remains a significant challenge. One promising approach is the implementation of digital twins for the design, simulation, and optimization of measurement processes within a virtual environment. In study [8], the aforementioned concepts were systematically defined, and a case study illustrating the application of selected solutions was presented. The terminology describing different measurement approaches was classified according to both the location and timing of the measurement process. Among them, in-process measurements, performed during machining, are widely applied in modern manufacturing systems [9,10]. Recent studies have demonstrated the application of proximity sensors [11], touch-trigger probes [12], and 3D scanners [13] for on-machine measurements. While contact systems provide reliable dimensional verification, optical methods remain sensitive to highly reflective surfaces and coolant contamination.

Thread measurement methods can be classified into contact and non-contact techniques, and numerous studies have investigated their automation and integration with machine tools [14,15]. An example of thread measurement performed directly within the machine tool environment is presented in [16]. The proposed solution achieved a measurement accuracy of approximately ±8 µm while maintaining a high inspection rate. The authors emphasized the potential of the method for rapid product verification. In [17,18], a measurement system based on a vision sensor mounted on a manipulator adjacent to the machine tool was developed. The obtained results showed good agreement with reference measurements performed using a microscope, with deviations not exceeding 6 µm. It should be noted, however, that these investigations were conducted under controlled experimental conditions, which significantly reduced the influence of disturbances and environmental factors typically encountered during actual machining operations. Consequently, the reported measurement performance may not fully reflect the accuracy achievable under real shop-floor conditions.

In the case of high-precision components produced for the aerospace industry [19], in-process measurements, although beneficial for process monitoring and quality assurance, are generally not recognized by certification standards as a substitute for final inspection, which remains the definitive verification of product dimensional conformity. One of the main reasons for this is the lower measurement accuracy achievable under production conditions compared with laboratory-based inspections, even when the same probing system is used, as demonstrated in [20]. Measurement deviations observed under production conditions originate from numerous sources, making the identification of their root causes a challenging task. To address this issue, researchers have developed mathematical models aimed at predicting and compensating for measurement errors [21]. Considerable attention has also been devoted to the estimation of measurement uncertainty, which represents one of the key challenges in industrial metrology. Various approaches to uncertainty evaluation in machine-integrated measurement systems have been discussed in the literature [22,23,24,25,26].

Environmental conditions significantly influence measurement accuracy. Temperature variations affect the thermal expansion of both the workpiece and machine tool, while coolants, lubricants, and chips may introduce additional random errors. Machine tool errors, including wear, collision damage, and axis positioning inaccuracies, also contribute to measurement uncertainty. Machines equipped with linear scales generally provide higher positioning accuracy than those using only rotary encoders. Consequently, machine geometry and positioning accuracy should be regularly verified [27,28,29]. The workpiece also contributes to measurement uncertainty through its rigidity, surface roughness [30], and thermomechanical deformation during machining [31]. Measurement uncertainty is additionally affected by the adopted measurement strategy and probe stylus selection, as inappropriate stylus geometry or dimensions may limit feature accessibility and introduce systematic errors. Probe performance is influenced by signal processing, transmission delay, stylus geometry, probing speed, and hysteresis [32,33,34]. Maintaining an appropriate probing speed and a consistent probing direction improves measurement repeatability. Although touch-trigger probes can also be applied to freeform surfaces [35], measurement errors increase considerably compared with two-dimensional features, with reported deviations exceeding 0.2 mm for complex geometries [36].

The present work addresses the automation of precision thread measurement for aerospace components. Despite the growing interest in on-machine metrology, dedicated solutions for the automatic measurement of precision threads are not currently offered by major machine tool or probing system manufacturers. In addition, only limited information is available in the scientific literature regarding the practical implementation of such measurements in industrial environments. Recent studies have focused primarily on non-contact thread inspection methods based on machine vision, laser sensors, and structured light systems, enabling the rapid online measurement of thread geometry. However, these approaches generally require dedicated external measuring systems and are not directly integrated into CNC machining cycles [37,38,39,40]. Therefore, the development of universal on-machine probing methods implemented directly within CNC controllers remains an important research direction. In particular, the application of contact-based probing systems for automated thread measurement directly on CNC machine tools has not been sufficiently investigated. Existing solutions based on touch-trigger or strain-gauge probes are mainly limited to standard dimensional inspections, while their application for precision thread evaluation remains an open research problem. To address this gap, the paper presents a methodology for measuring the pitch diameter of buttress threads using a contact-based strain-gauge probe mounted on a CNC machine tool. The proposed approach was validated through experiments conducted under actual production conditions. The results provide valuable information regarding the achievable measurement accuracy and repeatability, as well as the practical limitations associated with the implementation of automated thread inspection in aerospace manufacturing.

## 2. Materials and Methods

**Figure 1:**
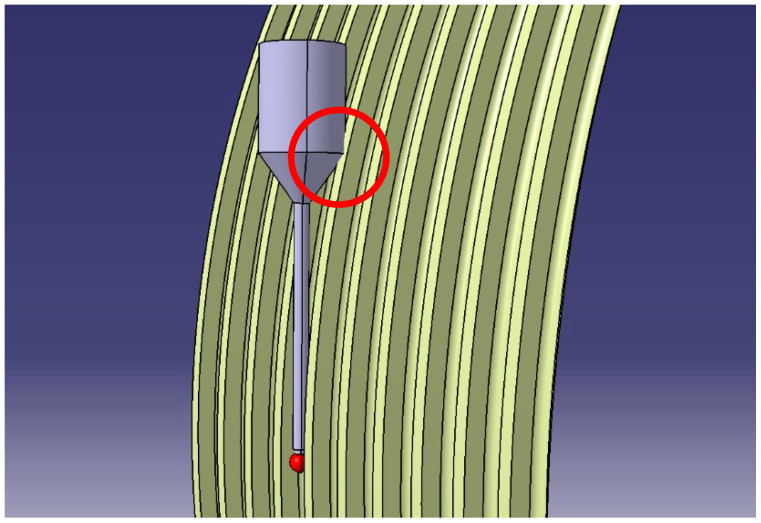
Example of a potential collision due to an excessively short stylus. The red circle highlights the area where the potential collision may occur.

The experimental tests were carried out on a WFL^®^ M40 MILLTURN machine tool (WFL Millturn Technologies GmbH & Co. KG, Linz, Austria). Based on comparative investigations, a Renishaw^®^ RMP600 strain-gauge touch-trigger probe (Renishaw, Gloucestershire, UK) was selected. The stylus ball diameters were chosen based on commercially available sizes corresponding as closely as possible to the diameter of the measuring wires used in the reference method. The feasibility of the selected probe configurations was verified using a three-dimensional model developed in the CATIA^®^ (V5R2020) environment (Dassault Systèmes, Vélizy-Villacoublay, France). The use of an excessively short probe stylus may lead to potential collisions or premature triggering of the probe signal due to unintended contact between the stylus shaft and the workpiece surface (Figure 1).

**Figure 2:**
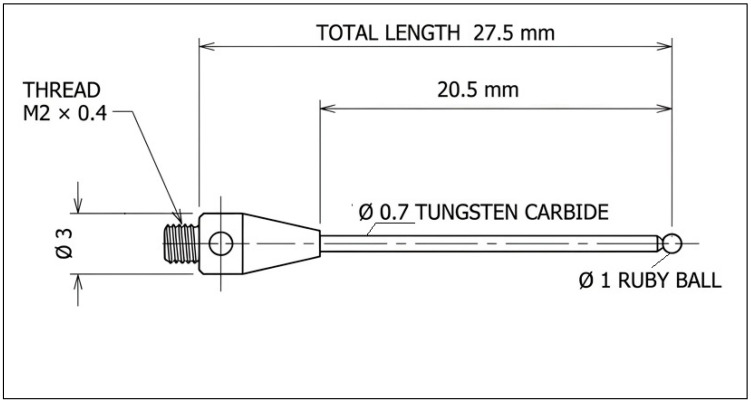
Drawing of the A-5000-8663 (Renishaw, UK) measuring tip [41].

The following assembly was configured from a total length of 107.50 mm, including the following components: extension L = 75 mm, reduction M3/M2, and a tip with a ruby ball of ϕ = 1 mm diameter (Figure 2).

**Figure 3:**
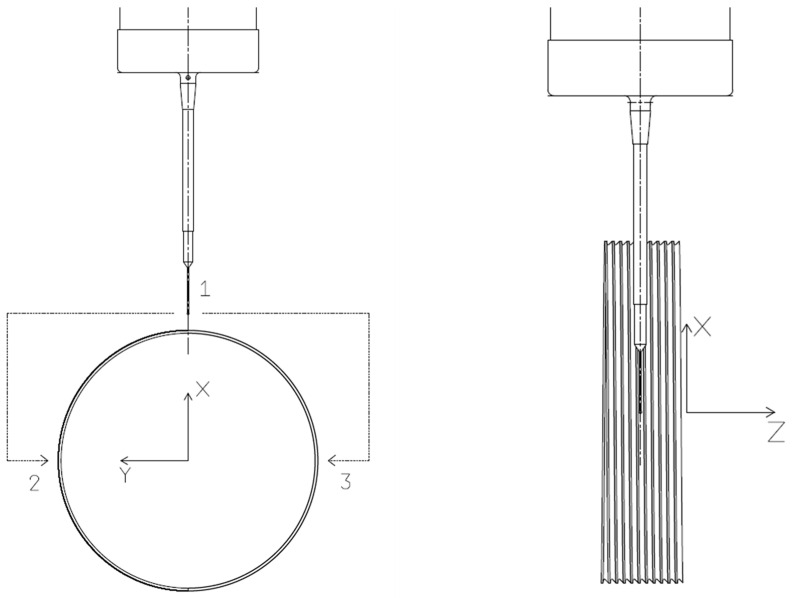
Schematic representation of the measurement path with machine-axis identification. (1) Initial approach position in the *Z*-axis direction; (2) and (3) measurement points in the *Y*-axis direction on opposite sides of the workpiece axis.

**Figure 4:**
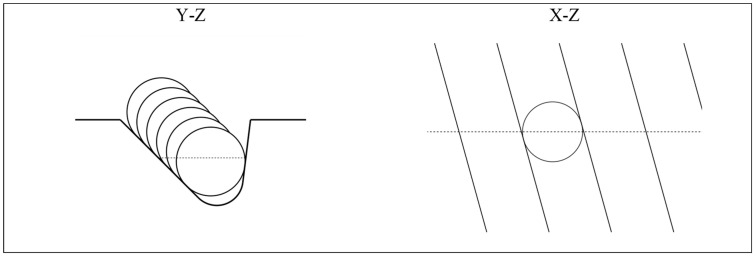
Schematic representation of the measuring tip within the thread profile.

To ensure maximum system rigidity, a stylus length of L = 20.5 mm was selected, corresponding to the shortest commercially available standard configuration. The stylus shaft was manufactured from carbide, while the stylus ball was made of ruby. A universal parametric measurement algorithm was developed and implemented as an NC program using R-variables within the Siemens SINUMERIK^®^ 840D control system (Siemens AG, München, Germany). The program was designed to allow adaptation to different thread types and diameter ranges through a limited set of input parameters, including thread pitch, nominal pitch diameter, expected probe output value, and the initial *Z*-axis position of the first measurement point. The measurement coordinate system was defined such that the probing direction was aligned along the Y+ and Y− axes, while the ball center was positioned at X = 0 during measurement. The measurement strategy involved an iterative search for the lowest point in the thread groove by incremental adjustment of the *Z*-axis position with a step of 0.01 mm. This step size was determined experimentally to ensure a compromise between measurement resolution and cycle time. A schematic representation of the toolpath and axis configuration is shown in Figure 3, while the position of the stylus ball within the thread profile is illustrated in Figure 4. The measurement in the Y–Z plane was performed on both thread flanks, with successive measurement points compared using logical evaluation of the recorded probe signals. Depending on the observed increase or decrease in the measured value, the subsequent probing point was shifted along the *Z*-axis in the appropriate direction. It should be noted that the actual contact between the stylus ball and the thread surface, due to the helix angle, occurs outside the Y–Z plane, as illustrated in the X–Z cross-sectional view.

**Figure 5:**
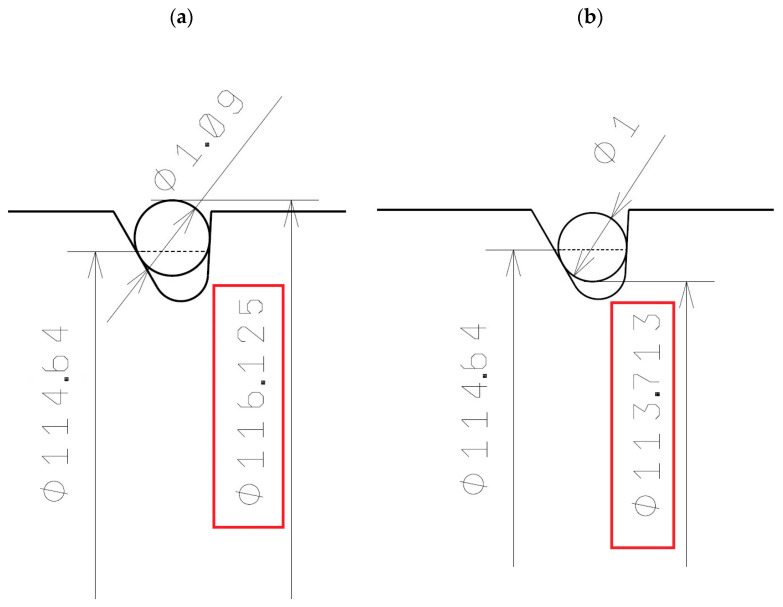
Example of indirect measurement of the pitch diameter of an external thread: (**a**) with the three-wire method; (**b**) with a touch-trigger probe. The red box indicates the measured diameter used for pitch diameter determination.

The measurement result was determined as the sum of the two minimum values obtained on both flanks of the shaft. The proposed approach is conceptually based on the two- and three-wire methods, which are widely regarded as the most accurate conventional techniques for measuring the pitch diameter of external threads. These methods rely on indirect measurement by exploiting the relationship between changes in pitch diameter and the corresponding variations in the measured over-wire dimension, obtained when measuring wires are positioned within the thread groove. In the case of probe-based measurement, the obtained value corresponds to the internal thread geometry, specifically to the contact point located on the side of the stylus ball closer to the root radius of the thread profile (Figure 5).

These values can also be determined using the Formula (1):

(1) $$M_{p}=d_{2}+d_{w}\left[1+\frac{\cos\left(\varepsilon \right)}{\sin\left(\frac{\alpha }{2}\right)}\right]-P\frac{\cos\beta *\cos\gamma }{\sin\alpha }+p_{1}+p_{2}$$

where d_2_—pitch diameter, d_v_—roller diameter, α = β + γ—thread angle, β; γ—flank angles, ε = (β + γ)/2—half-angle of the thread profile, P—pitch, p_1_—correction for the twisting of the rollers in the thread grooves, and p_2_—correction for elastic deformation caused by the measuring force acting between the rollers and the thread at the contact points.

To verify that the developed procedure meets the required performance criteria and provides reliable measurement results, a validation study was carried out. Due to the nature of the task and the availability of the machine tool, the experiments were performed under production (shop-floor) conditions. The presented results were obtained from single measurements performed on production-line components. The repeatability of the developed algorithm was verified separately by conducting 30 repeated measurements of the same thread position, confirming the stability of the proposed method. Following initial tests indicating high repeatability and stability of the proposed method, the measurement algorithm was subsequently integrated directly into the machining program. During shaft manufacturing, the NC program included a programmed pause to enable pitch diameter measurement using the three-wire method performed by a qualified operator. Prior to the pause, an automatic probe-based measurement was carried out, and the results were stored in the machine controller memory. Following the manual measurement, the operator recorded the results obtained by both methods in a dedicated data table. The measurements were collected for shafts produced in response to ongoing production demand. Four types of external threads with an asymmetric trapezoidal profile were selected for analysis and designated as THREAD1, THREAD2, THREAD3, and THREAD4. The geometrical parameters of the investigated threads are presented in Table 1.

The investigated threads differed in both nominal diameter and flank angle. The experiments were carried out on six machine tools designated as WFL1, WFL2, WFL3, WFL4, WFL5, and WFL6. The collected results were subjected to preliminary statistical analysis to identify and remove outliers, as well as to assess the normality of the data distribution. The dataset was grouped according to machine type and thread type. In the first stage, outliers exceeding the ±3σ criterion were excluded from further analysis.

A total of 1043 measurements were subsequently divided into groups according to thread type and machine tool type.

Based on these criteria, the number of repetitions in each group is as follows:

Thread type:

- THREAD 1—260 repetitions.
- THREAD 2—415 repetitions.
- THREAD 3—190 repetitions.
- THREAD 4—178 repetitions.

Type of machine:

- WFL1—118 repetitions.
- WFL2—177 repetitions.
- WFL3—151 repetitions.
- WFL4—260 repetitions.
- WFL5—73 repetitions.
- WFL6—264 repetitions.

Notably, threads with flank angles of 3°/30° were manufactured on machine tools WFL3, WFL4, and WFL6, whereas threads with angles of 7°/45° were produced on WFL1, WFL2, and WFL5.

To evaluate the accuracy of the developed method, the absolute error for each measurement pair (touch-trigger probe and three-wire method) was calculated according to the following relationship (2):

(2) $$\Delta x=x-x_{0}$$

where x—result obtained using a measurement probe, and x_0_—result obtained with the three-wire method.

Manual measurement using the three-wire method was adopted as the reference technique. As noted previously, this method is widely regarded as one of the most accurate and commonly used approaches for pitch diameter determination. Accordingly, the results obtained using this method were treated as reference values for the pitch diameter. The values obtained with the touch-trigger probe were considered measured values, and the corresponding measurement errors were subsequently evaluated.

Statistical analysis of the results was performed using Microsoft Excel^®^ software (Microsoft Corporation, Redmond, WA, USA). Basic statistical parameters, including the arithmetic mean, standard deviation, and range, were calculated. In addition, the Shapiro–Wilk test, one-way ANOVA, and Tukey’s HSD post hoc test were applied.

These analyses were used to assess the repeatability of the developed procedure across a large number of samples and, importantly, to identify potential differences related to both thread type and machine tool. The investigated machine tools, operating within the same production line, were grouped into a single machine set and were therefore assumed to be subjected to similar environmental conditions. During the experiments, the ambient temperature ranged from 23 °C to 26 °C, and the production hall was equipped with an air-conditioning system.

**Figure 6:**
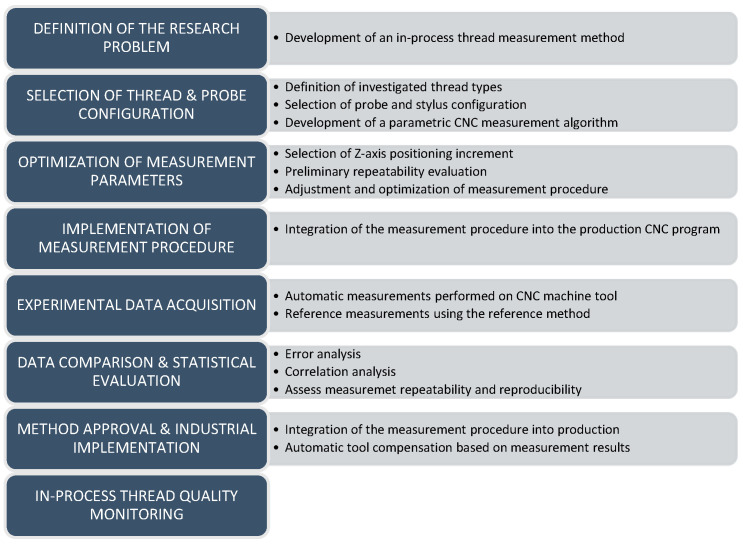
Flowchart of the developed measurement methodology and experimental validation procedure.

For improved clarity and better visualization of the experimental procedure, the overall workflow of the research conducted is presented in Figure 6. The flowchart summarizes the main stages of the developed measurement methodology, from initial configuration selection to industrial implementation.

## 3. Results and Discussion

To assess the distribution of the absolute errors, the Shapiro–Wilk test was applied. The null hypothesis of this test assumes that the sample originates from a normally distributed population. A statistically significant result (*p* < 0.05) indicates a deviation from normality.

The results are shown in the table below (Table 2).

For a significance level of α = 0.05, all test samples satisfied the condition *p* > α; therefore, there were no grounds to reject the null hypothesis. It can thus be concluded, based on the Shapiro–Wilk test, that the absolute error distributions do not deviate significantly from normality.

**Figure 7:**
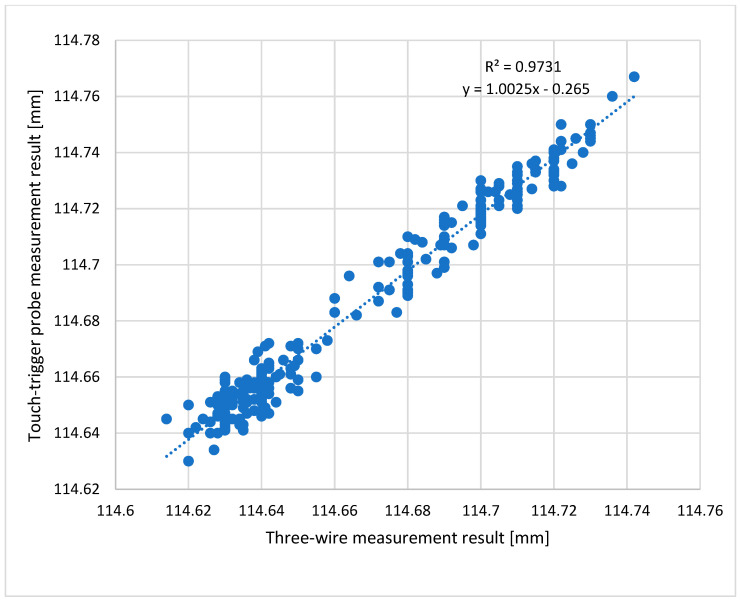
Correlation chart of the three-wire method with a probe for measuring the pitch diameter of THREAD1.

To further assess the relationship between the two measurement methods, the coefficient of determination (R^2^) was calculated. This coefficient indicates the proportion of variance in the dependent variable that is explained by the independent variable. The results for THREAD1 are presented graphically in Figure 7.

The relationship between the reference and probe-based measurements was found to be linear, confirming a strong correlation between the two methods. Moreover, the coefficient of determination was close to unity. The lowest R^2^ value of 0.9605 was obtained for THREAD2, indicating that approximately 96% of the variability in the probe-based measurements can be explained by the reference method. In practical terms, this suggests that a linear regression model can be used to predict reference values from probe measurements with high accuracy.

By partitioning the dataset into subgroups according to thread type and machine tool number, the arithmetic mean, median, standard deviation, maximum and minimum values, and the range of the obtained errors were calculated. The results are summarized in Table 3 and Table 4.

**Figure 8:**
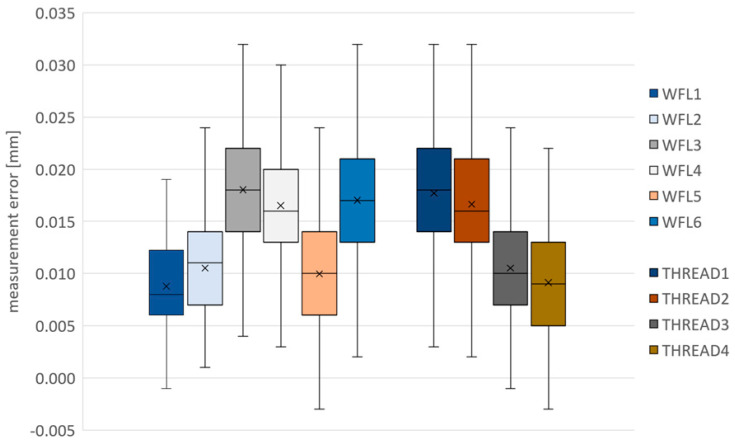
Measurement error distribution for different machines and thread types.

The arithmetic mean of the errors was lowest for THREAD4 (0.009 mm), while the highest mean value was observed for THREAD1 (0.018 mm), approximately twice as large. This indicates a systematic tendency for the probe-based measurements to yield slightly higher values compared to the reference results obtained using the three-wire method. The median values, due to the near-symmetric distribution of the data, were close to the corresponding arithmetic means. The standard deviation, representing the dispersion of results within each group, was approximately 0.006 mm. This relatively low value indicates a high concentration of measurements around the mean, which may confirm the repeatability of the proposed procedure. The range between the maximum and minimum values was approximately 0.03 mm for all groups. For comparison, the results are presented in the form of a box plot in Figure 8. 

**Figure 9:**
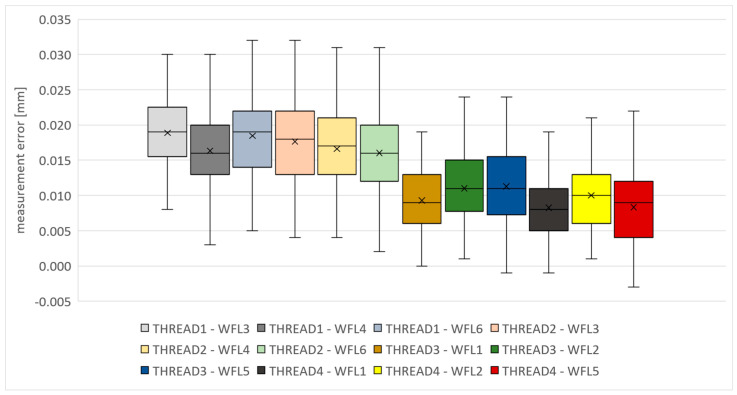
Detailed measurement error graph for thread–machine combinations.

The first six bars from the left correspond to machine tool types, whereas the remaining four refer to thread types. Visually, differences in the value obtained can be observed. The mean errors for the first three machine tools are slightly lower, at approximately 0.008 mm, compared to the remaining three machines, for which an average error of approximately 0.017 mm was recorded. A similar trend is observed for individual thread types, where THREAD1 and THREAD2 exhibit higher errors than THREAD3 and THREAD4. This observation is related to the fact that THREAD3 and THREAD4 were produced and measured on WFL1, WFL2, and WFL5 machines, whereas THREAD1 and THREAD2 were produced and measured on WFL3, WFL4, and WFL6 machines. Differences in measurement error may therefore be associated with both machine tool characteristics and the implementation of the measurement algorithm. The largest spread in results, defined as the difference between the maximum and minimum values, was observed for THREAD2, with a range of 0.03 mm. The previously discussed results were based on aggregated data from multiple machines and thread types. To further investigate the influence of these factors and reduce potential confounding effects, the dataset was disaggregated. The resulting analysis is presented in Figure 9, which provides a more detailed comparison of the individual cases.

In the graph, variations are evident not only between THREAD1/THREAD2 and THREAD3/THREAD4, but also among individual machine tools. For instance, the bar corresponding to THREAD1 on machine WFL4 exhibits slightly lower values compared to WFL3 and WFL6 for the same thread type. For THREAD3 and THREAD4, the differences are more pronounced.

To analyze differences in mean errors between the studied groups, an ANOVA test was performed. The assumptions of this test include normality of the residuals and homogeneity of variances across groups. Normality was confirmed using the Shapiro–Wilk test, while Levene’s test indicated that the assumption of homogeneity of variances was satisfied.

Table 5 and Table 6 present the results of the analysis of variance for THREAD1/THREAD2 and THREAD3/THREAD4, respectively. In both cases, the F-statistic exceeded the critical value and the *p*-value was below 0.05, leading to rejection of the null hypothesis and acceptance of the alternative hypothesis, which states that at least one group mean differs significantly from the others. However, for THREAD3 and THREAD4, the *p*-value was close to the significance level α = 0.05, indicating marginal statistical significance.

To identify specific group differences, a Tukey HSD post hoc test was conducted. The results are presented in Table 7 and Table 8.

When comparing THREAD1 and THREAD2 across different machine tools, no statistically significant differences were found for the same thread type measured on different machines. However, the post hoc analysis identified two cases in which the mean values differed noticeably from the remaining comparisons. These correspond to the pairs THREAD1–WFL3 versus THREAD2–WFL6 and THREAD1–WFL6 versus THREAD2–WFL6. It should be noted that these comparisons involve different thread types. Moreover, in the second case, the Tukey HSD test indicated significant differences within the WFL6 machine data.

Overall, these results suggest that the influence of the machine tool on measurement discrepancies is limited, which may indicate both the high stability of the measurement system and the satisfactory technical condition of the machine tools and probes.

The observed differences in the ANOVA and post hoc analyses appear to be more strongly associated with the measurement algorithm than with the machine tools themselves. The differences in mean error values for the identified pairs range from 0.002 to 0.003 mm, which is relatively small. Nevertheless, the results indicate the presence of minor variability, suggesting that a more tailored approach for individual thread types may be beneficial.

In the analysis of THREAD3 and THREAD4 samples, the *p*-value was close to the significance threshold; nevertheless, the post hoc test also identified two pairs exhibiting noticeable deviations from the remaining comparisons. These correspond to THREAD3–WFL2 versus THREAD4–WFL1 and THREAD3–WFL5 versus THREAD4–WFL1. As in previous cases, these differences are primarily associated with the thread type. The differences in mean values between the analysed groups are approximately 0.003 mm.

An attempt was made to explain the observed discrepancies and, in particular, to identify the sources of the repeatable measurement errors, which may be classified as systematic errors. The largest differences were observed in the determination of pitch diameter for threads with flank angles of 3°/30° and 7°/45°, indicating that the flank angle has a significant influence on the obtained measurement results.

When external and environmental factors are neglected, and assuming a correctly calibrated probe and properly aligned machine tool, the observed discrepancies are likely related to the measurement algorithm. The measurement strategy itself, which involves logical comparison of successive measured points to locate the minimum position along the *Y*-axis, appears to be consistent, as no significant differences were observed between repeated trials on the same machine.

A critical factor influencing the obtained results is the *Z*-axis positioning during successive measurements. The theoretical two-point measurement is difficult to achieve in practice due to the kinematics of the touch-trigger probe. Consequently, the measured point used for determining the tool offset correction depends on the *Z*-axis displacement of the probe.

**Figure 10:**
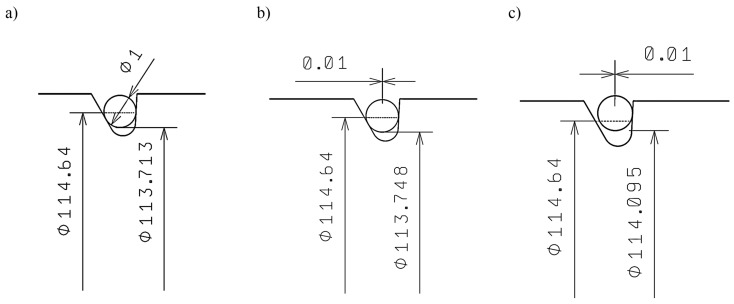
Example of the influence of the probe shift on the results obtained when measuring the THREAD1. (**a**) ideal two-point contact of the probe stylus with both thread flanks; (**b**) probe stylus shifted 0.01 mm to the left; (**c**) probe stylus shifted 0.01 mm to the right.

For example, in the case of THREAD1, as illustrated in Figure 10, the left side of Figure 10a shows the ideal situation where the probe tip makes contact with both sides of the notch. In this ideal scenario, the predicted pitch diameter from the measuring probe would be ϕ = 113.713 mm. However, if the probe is moved 0.01 mm towards the side with a 30° angle, the result is ϕ = 113.748 mm. Conversely, moving the probe towards the side with a 3° angle results in a measurement of ϕ = 114.095 mm. In this case, the measurement would be +0.035 mm greater for the 30° side and +0.382 mm greater for the 3° side.

**Figure 11:**
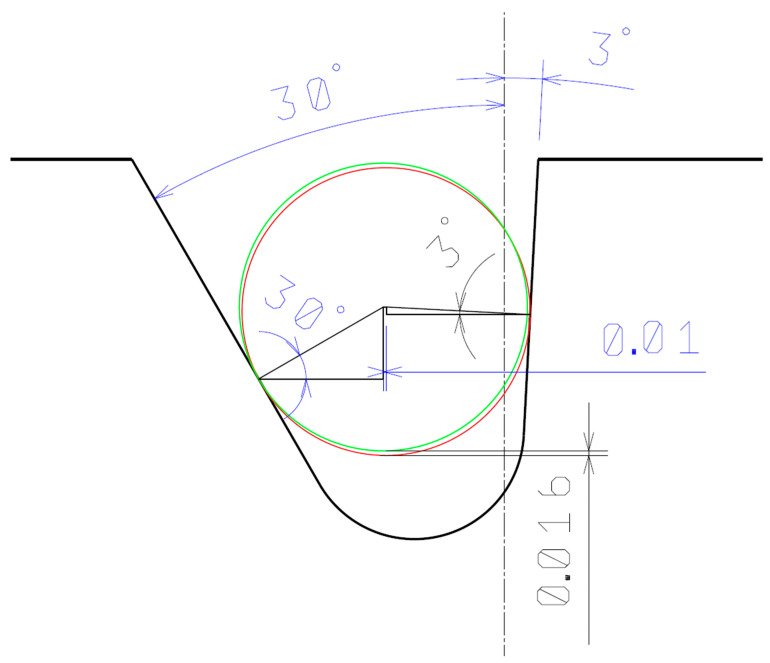
An auxiliary sketch to determine the measurement error resulting from the probe positioning accuracy of 0.01 mm.

It can be assumed that a deviation of nearly 0.4 mm would be detected by the logical comparison criteria; however, the actual error resulting from positioning accuracy must be evaluated using a different approach. This is because it is still possible to reach the lowest measuring point on the flank with a 3° angle. To determine the interval within which the point used for diameter calculation is collected, a schematic representation was developed (Figure 11).

**Figure 12:**
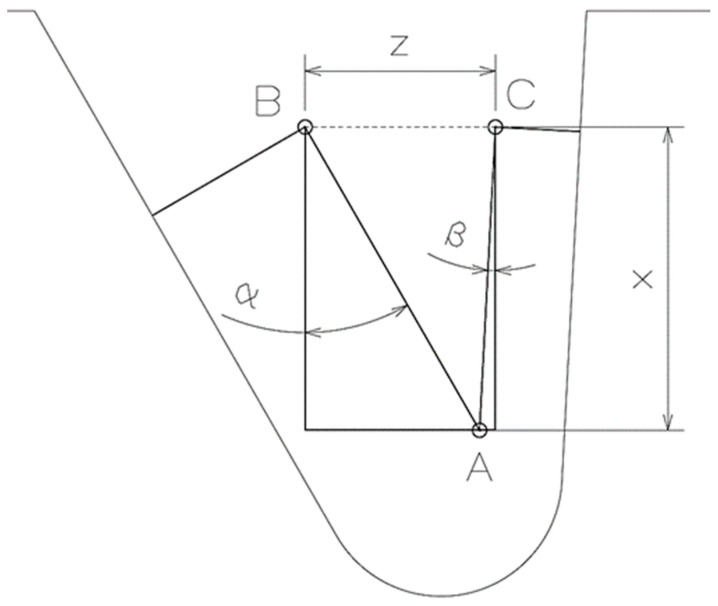
An auxiliary sketch for determining the overall error resulting from the “z” positioning accuracy.

To determine the actual interval within which the final measurement point is obtained, two circles (green and red) with a diameter equal to that of the measuring sphere were constructed. Each circle was made tangent to one flank of the groove profile. The blue circle represents the ideal situation in which the measuring sphere is located at the lowest point and simultaneously contacts both flanks of the thread profile. The distance between the centers of green and red circles was defined as the positioning increment, i.e., 0.01 mm. Importantly, the centers of the circles were positioned at the same height, meaning that each configuration was defined such that the controller would register the same contact condition after probe engagement. Under these conditions, the measured value at the extreme positions would be 0.016 mm higher than in the ideal case, i.e., two-point contact. Considering that the pitch diameter is determined as a two-sided measurement, the resulting deviation would be approximately twice as large, i.e., about 0.032 mm. This value corresponds well to the maximum error observed for THREAD1 and THREAD2, which correspond to thread profiles with 3°/30° flank angles. For arbitrary flank angles, a general mathematical relationship can be derived to describe the maximum error resulting from the *Z*-axis positioning resolution. The corresponding relationships are illustrated in Figure 12.

Point A defines the center of the circle in the ideal position, whereas points B and C represent the centers of the circles in the extreme positions, depending on the positioning increment z. Angles α and β correspond to the flank angles of the thread profile.

Assuming that the segment z is divided into two parts, a and b, the following relationship is obtained:

(3) z=a+b

(4) $$tg\;\alpha =\frac{a}{x}$$

(5) $$tg\;\beta =\frac{b}{x}$$

(6) z=a+b=x tg α+x tg β=x(tg α+tg β)

Taking into account the tested threads, the following relationship is obtained:

(7) $$x=\frac{z}{\left(tg\;\alpha +tg\;\beta \right)}=\frac{0.01}{tg30^{\circ }+tg3^{\circ }}\approx 0.016\;mm$$

(8) $$x=\frac{0.01}{tg45^{\circ }+tg7^{\circ }}\approx 0.009\;mm$$

These values represent the maximum error relative to the shaft axis. To obtain the corresponding diameter errors, the values must be doubled, resulting in 0.023 mm and 0.018 mm. This amplification explains the differences observed between results obtained for the two thread flank angle configurations.

The study indicates that the measurement accuracy errors are repeatable and partially predictable. Reduction in these errors may be achieved by decreasing the *Z*-axis positioning step, as previous analysis has shown this parameter to be critical. However, reducing the step size, for example to 0.005 mm, would increase the overall measurement time. In thread measurement applications, a trade-off must therefore be considered between measurement accuracy and cycle time, particularly since final conformity of the manufactured part is verified using a separate inspection method.

Although the statistical analysis confirmed the repeatability and consistency of the proposed measurement method, the practical applicability of the results also depends on the associated measurement uncertainty. Therefore, an uncertainty analysis was carried out to quantify the confidence in the measurement results obtained.

In order to estimate the measurement uncertainty of the proposed method, the expanded uncertainty U was determined using the following relationship:

(9) $$U=k\;\cdot \;u_{c}$$

where u_c_—combined standard uncertainty and k—coverage factor, assumed to be equal to 2.

Due to the large number of factors affecting the measurement result, the uncertainty budget was limited to the most critical contributions. To investigate the influence of the measurement system, a test was performed using a Class I gauge block with a length of L = 100 mm. This length is close to the pitch diameter of the measured threads. A series of 50 two-point measurements of the reference artefact was carried out using the measuring probe along the *Y*-axis direction. The gauge block had been kept in the machine environment for an extended period, and the machine tool was thermally stabilized immediately after the production part had been completed.

The measurement uncertainty of the investigated measurement system was evaluated for the gauge block according to the following relationship [26]:

(10) $$u_{cp}=\;\sqrt{u_{Ap}^{2}+u_{L}^{2}+u_{R}^{2}}$$

where $u_{cp}$—combined standard uncertainty for the gauge block, $u_{Ap}$—standard uncertainty evaluated using the Type A method for the gauge block, $u_{L}$—uncertainty associated with the gauge block length, and $u_{R}$—uncertainty contribution due to the resolution of the measurement system.

The standard uncertainty was estimated using the Type A evaluation method. It was assumed that u_Ap_ is equal to the standard deviation of the series of 50 repeated measurements, resulting in u_Ap_ = 0.6 μm.

The standard uncertainty associated with the gauge block length was evaluated using a Type B evaluation method, assuming a rectangular probability distribution.

(11) $$u_{L}=\frac{t_{e}}{\sqrt{3}}=\frac{0.6}{\sqrt{3}}\approx 0.4\;\mu m$$

where t_e_—maximum permissible deviation from the nominal length of the gauge block. For a Class I gauge block with a length of L = 100 mm, the tolerance limit *t_e_* is assumed to be ±0.6 μm.

The standard uncertainty due to the resolution of the measurement system was also evaluated using a Type B method, assuming a triangular probability distribution.

(12) $$u_{R}=\frac{0.05}{\sqrt{6}}\approx 0.2\;\mu m$$

The combined standard uncertainty of the measurement system can therefore be evaluated using the following relationship:

(13) $$u_{cp}=\;\sqrt{u_{Ap}^{2}\;+u_{L}^{2}\;+u_{R}^{2}}\approx 0.8\;\mu m$$

The calculated measurement uncertainty value refers, in a simplified manner, to the measuring equipment consisting of the machine tool and the touch-trigger probe.

The uncertainty budget for the investigated measurement procedure of the external thread pitch diameter should also include the geometry of both the measuring tip and the measured thread.

Due to the similarity of the proposed measurement method to the three-wire measurement method, the uncertainty of the investigated system can be estimated using the equation dedicated to this method [3]:

(14) $$u_{c}=\sqrt{u^{2}\left(M_{p}\right)\;+u^{2}\left(d_{w}\right)\;+u^{2}\left(P\right)\;+u^{2}\left(\beta \right)\;+u^{2}\left(\gamma \right)\;+u^{2}\left(p_{1}\right)\;+u^{2}\left(p_{2}\right)}$$

where the contributions of the measurement uncertainty budget correspond to:

1. The measurement system contribution (evaluated based on measurements of the gauge block):
  (15) $$u\left(M_{p}\right)\;=u_{cp}=0.748\;\mu m$$
2. The contribution of the measuring wire (measuring ball) diameter:
  (16) $$u\left(d_{w}\right)\;=\left(1\;+\;\frac{\cos\varepsilon }{\sin\frac{\alpha }{2}}\right)$$
3. The contribution of the thread pitch:
  (17) $$u\left(P\right)\;=\;\frac{\cos\beta \cos\gamma }{\sin\alpha }$$
4. The contribution of the thread profile half-angle:
  (18) $$u\left(\beta \right)=\frac{0.1454}{{sin}^{2}\frac{\alpha }{2}}\cos\beta (d_{w}-d_{o1})$$
  (19) $$u\left(\gamma \right)=\frac{0.1454}{{sin}^{2}\frac{\alpha }{2}}\cos\gamma (d_{w}-d_{o2})$$
5. The correction contribution due to measuring wire deformation:
  (20) $$u(p_{1})$$
6. The contribution due to the measuring force correction:
  (21) $$u(p_{2})$$

The uncertainty contribution associated with temperature effects was not included in the calculations.

Although statistically significant differences were observed between some measurement groups, their practical significance is limited. The average difference between the proposed method and the reference three-wire method did not exceed approximately ±0.015 mm remains substantially lower than the specified tolerance. Furthermore, the expanded measurement uncertainty of the proposed method (6.7–9.5 μm), which corresponds to about 7% of the 140 μm tolerance field for the investigated pitch diameter. These results indicate that the proposed measurement methodology provides sufficient accuracy for industrial inspection and process control of the investigated buttress threads.

In aerospace applications, each thread undergoes a final functional test to identify non-conforming parts. Consequently, the proposed measurement method should be considered an auxiliary tool for automatic tool offset correction and for preliminary verification of whether further machining adjustments are required, especially since such corrections are difficult to implement once the shaft has been removed from the machine tool. Corrective actions may be implemented using mathematical models and algorithmic compensation to account for systematic errors.

The general applicability of the proposed method depends mainly on the geometric relationship between the thread profile and the probe configuration. For reliable pitch diameter measurement, the probe sphere must establish a two-point contact condition close to the pitch diameter region. Therefore, the selection of probe diameter and thread size represents an important constraint for extending the method to other thread standards.

## 4. Conclusions

The conducted study, based on measurements of over 1000 workpieces, demonstrated the feasibility of repeatable pitch diameter measurement of asymmetric trapezoidal threads directly on a CNC machine tool. The proposed measurement procedure was validated against a reference method based on the three-wire technique, demonstrating a high level of linear correlation between the two approaches.

The developed measurement procedure demonstrated a satisfactory level of accuracy and repeatability, indicating a systematic tendency for probe-based measurements to yield slightly higher values compared to the reference method. The standard deviation of approximately 0.006 mm and the overall range of about 0.03 mm.

Statistical evaluation, including ANOVA and Tukey’s HSD post hoc test revealed that, while some statistically significant differences exist between selected groups, these are primarily associated with the type of thread rather than the machine tool used. Box plot analysis further confirmed that thread geometry has a greater influence on measurement variation than differences between machining centers.

The results indicate that the thread flank angle has a significant influence on measurement accuracy. Threads with different flank angles (3°/30° and 7°/45°) exhibited noticeable differences in the obtained results. The *Z*-axis positioning step was also identified as a relevant factor, suggesting that finer increments may reduce measurement error, although at the cost of increased measurement time.

The method enables real-time measurement and automatic correction during production, supporting efficient and reliable thread evaluation in industrial environments. However, depending on application requirements, limited manual verification or adjustment may still be necessary.

Further improvements in accuracy may be achieved by reducing the *Z*-axis positioning step, provided that the resulting increase in cycle time is acceptable. Future work may also focus on optimization of the measurement algorithm to reduce systematic errors and to improve robustness across different thread profiles and material conditions.

Overall, the results confirm that CNC machine tools, although not certified metrology systems, can be effectively used as auxiliary devices for automated preliminary inspection and process monitoring.

## Figures and Tables

**Table 1:** List of the most important dimensions of the tested threads [42,43].

	THREAD1	THREAD2	THREAD3	THREAD4
Type by ANSI B1.9 [42]:	4.5590-12 BUTT-3A	4.1098-12 BUTT-3A	4.1150-10 BUTT-3A	3.7668-10 BUTT-3A
Pitch per inch	12	12	10	10
Pitch [mm]	2.11	2.11	2.54	2.54
Pitch diameter [mm]	Ø = 114.64 ± 0.07	Ø = 103.165 ± 0.07	Ø = 103.335 ± 0.07	Ø = 94.518 ± 0.07
Three-wire measurement [mm]	Ø = 116.12 ± 0.07	Ø = 104.638 ± 0.07	Ø = 104.933 ± 0.07	Ø = 96.114 ± 0.07
Diameter of wires [mm]	Ø = 1.09	Ø = 1.09	Ø = 1.22	Ø = 1.22
Probe measurement Ø = 1 [mm]	Ø = 113.713 ± 0.07	Ø = 102.238 ± 0.07	Ø = 102.229 ± 0.07	Ø = 93.412 ± 0.07
Major diameter [mm]	Ø = 115.8 ± 0.07	Ø = 104.318 ± 0.07	Ø = 104.445 ± 0.07	Ø = 95.606 ± 0.07
Minor diameter [mm]	Ø = 113.276 max	Ø = 101.709 max	Ø = 101.983 max	Ø = 93.167 max
Root radius [mm]	R0.394 ± 0.037	R0.394 ± 0.037	R0.394 ± 0.037	R0.394 ± 0.037
Lead flank angle	3°	3°	7°	7°
Clearance flank angle	30°	30°	45°	45°

**Table 2:** Shapiro–Wilk test results.

	W-Stat	*p*-Value	α	*p* > α
THREAD1–WFL3	0.9771	0.4498	0.05	yes
THREAD1–WFL4	0.9844	0.2646	0.05	yes
THREAD1–WFL6	0.9866	0.3612	0.05	yes
THREAD2–WFL3	0.9844	0.2723	0.05	yes
THREAD2–WFL4	0.9897	0.3115	0.05	yes
THREAD2–WFL6	0.9910	0.4280	0.05	yes
THREAD3–WFL1	0.9699	0.1439	0.05	yes
THREAD3–WFL2	0.9814	0.2273	0.05	yes
THREAD3–WFL5	0.9748	0.5040	0.05	yes
THREAD4–WFL1	0.9658	0.1005	0.05	yes
THREAD4–WFL2	0.9796	0.1846	0.05	yes
THREAD4–WFL5	0.9794	0.7675	0.05	yes

**Table 3:** Analysis of measurement errors by thread type [43].

[mm]	THREAD1	THREAD2	THREAD3	THREAD4
Mean value	0.018	0.017	0.011	0.009
Median	0.018	0.016	0.010	0.009
Std. dev	0.006	0.006	0.005	0.005
Min.	0.003	0.002	−0.001	−0.003
Max.	0.032	0.032	0.024	0.022
Range	0.029	0.030	0.025	0.025

**Table 4:** Analysis of measurement errors divided by machine number [43].

[mm]	WFL1	WFL2	WFL3	WFL4	WFL5	WFL6
Mean value	0.009	0.011	0.018	0.017	0.010	0.017
Median	0.008	0.011	0.018	0.016	0.010	0.017
Std. dev	0.005	0.005	0.006	0.006	0.006	0.006
Min.	−0.001	0.001	0.004	0.003	−0.003	0.002
Max.	0.019	0.024	0.032	0.031	0.024	0.032
Range	0.020	0.023	0.028	0.028	0.027	0.030

**Table 5:** Results of the ANOVA test for samples from the THREAD1 and THREAD2 groups.

One-Way ANOVA						
Summary						
Groups	Count	Sum	Mean	Variance		
THREAD1–WFL3	49	0.9250	0.0189	0.00003		
THREAD1–WFL4	104	1.7000	0.0163	0.00004		
THREAD1–WFL6	107	1.9770	0.0185	0.00004		
THREAD2–WFL3	102	1.8000	0.0176	0.00004		
THREAD2–WFL4	156	2.5920	0.0166	0.00003		
THREAD2–WFL6	157	2.5170	0.0160	0.00003		
Analysis of variance						
Source of variance	SS	df	MS	F	*p*-value	Test F
Between groups	0.0007	5	0.0001	3.7061	0.0026	2.2275
Within groups	0.0239	669	0.0000			
Total	0.0245	674				

where: SS—sum of squares; df—degrees of freedom; MS—mean square; F—F statistic; Test F—critic F value.

**Table 6:** Results of the ANOVA test for samples from the THREAD3 and THREAD4 groups [43].

One-Way ANOVA						
Summary						
Groups	Count	Sum	Mean	Variance		
THREAD3–WFL1	60	0.5570	0.0093	0.00003		
THREAD3–WFL2	90	0.9900	0.0110	0.00003		
THREAD3–WFL5	40	0.4520	0.0113	0.00003		
THREAD4–WFL1	58	0.4780	0.0082	0.00002		
THREAD4–WFL2	87	0.8710	0.0100	0.00002		
THREAD4–WFL5	33	0.2750	0.0083	0.00004		
Analysis of variance						
Source of variance	SS	df	MS	F	*p*-value	Test F
Between groups	0.0005	5	0.0001	3.4151	0.0050	2.2389
Within groups	0.0096	362	0.0000			
Total	0.0100	367				

**Table 7:** Results of the Tukey HSD test for samples from the THREAD1 and THREAD2 groups.

TUKEY HSD/KRAMER									
Groups	Mean	Count	ss	df	q-crit	significance level α =0.05			
THREAD1–WFL3	0.0189	49	0.0013						
THREAD1–WFL4	0.0163	104	0.0038						
THREAD1–WFL6	0.0185	107	0.0040						
THREAD2–WFL3	0.0176	102	0.0043						
THREAD2–WFL4	0.0166	156	0.0052						
THREAD2–WFL6	0.0160	157	0.0053						
		675	0.0239	669	4.03				
**Q TEST**									
group 1	group 2	mean	std. dev.	q statistic	lower bound of the confidence interval	upper bound of the confidence interval	*p*-value	critical difference of means	effect size (Cohen’s d)
THREAD1–WFL3	THREAD1–WFL4	0.0025	0.0007	3.4579	−0.0004	0.0055	0.1424	0.0030	0.4237
THREAD1–WFL3	THREAD1–WFL6	0.0004	0.0007	0.5501	−0.0025	0.0033	0.9988	0.0029	0.0671
THREAD1–WFL3	THREAD2–WFL3	0.0012	0.0007	1.6756	−0.0017	0.0042	0.8441	0.0030	0.2059
THREAD1–WFL3	THREAD2–WFL4	0.0023	0.0007	3.2696	−0.0005	0.0051	0.1906	0.0028	0.3786
THREAD1–WFL3	THREAD2–WFL6	0.0028	0.0007	4.1162	0.0001	0.0056	0.0431	0.0028	0.4763
THREAD1–WFL4	THREAD1–WFL6	0.0021	0.0006	3.6621	−0.0002	0.0045	0.1012	0.0023	0.3566
THREAD1–WFL4	THREAD2–WFL3	0.0013	0.0006	2.2096	−0.0011	0.0037	0.6237	0.0024	0.2177
THREAD1–WFL4	THREAD2–WFL4	0.0003	0.0005	0.5034	−0.0019	0.0024	0.9993	0.0022	0.0451
THREAD1–WFL4	THREAD2–WFL6	0.0003	0.0005	0.5884	−0.0018	0.0025	0.9984	0.0022	0.0526
THREAD1–WFL6	THREAD2–WFL3	0.0008	0.0006	1.4189	−0.0015	0.0032	0.9168	0.0024	0.1388
THREAD1–WFL6	THREAD2–WFL4	0.0019	0.0005	3.5097	−0.0003	0.0040	0.1310	0.0021	0.3115
THREAD1–WFL6	THREAD2–WFL6	0.0024	0.0005	4.6160	0.0003	0.0046	0.0146	0.0021	0.4092
THREAD2–WFL3	THREAD2–WFL4	0.0010	0.0005	1.9177	−0.0011	0.0032	0.7533	0.0022	0.1727
THREAD2–WFL3	THREAD2–WFL6	0.0016	0.0005	3.0062	−0.0006	0.0038	0.2752	0.0022	0.2703
THREAD2–WFL4	THREAD2–WFL6	0.0006	0.0005	1.2218	−0.0013	0.0025	0.9549	0.0019	0.0977

where: ss—Sum of squares; df—Degrees of freedom; q-crit—Critical q value.

**Table 8:** Results of the Tukey HSD test for samples from the THREAD3, THREAD4 groups.

TUKEY HSD/KRAMER									
Groups	Mean	Count	ss	df	q-crit	significance level α =0.05			
THREAD3–WFL1	0.0093	60	0.0015						
THREAD3–WFL2	0.0110	90	0.0023						
THREAD3–WFL5	0.0113	40	0.0013						
THREAD4–WFL1	0.0082	58	0.0012						
THREAD4–WFL2	0.0100	87	0.0020						
THREAD4–WFL5	0.0083	33	0.0012						
		368	0.0096	362	4.0519				
**Q TEST**									
group 1	group 2	mean	std. dev.	q statistic	lower bound of the confidence interval	upper bound of the confidence interval	*p*-value	critical difference of means	effect size (Cohen’s d)
THREAD3–WFL1	THREAD3–WFL2	0.0017	0.0006	2.8355	−0.0007	0.0042	0.3415	0.0025	0.3342
THREAD3–WFL1	THREAD3–WFL5	0.0020	0.0007	2.7197	−0.0010	0.0050	0.3896	0.0030	0.3926
THREAD3–WFL1	THREAD4–WFL1	0.0010	0.0007	1.5577	−0.0017	0.0038	0.8806	0.0027	0.2028
THREAD3–WFL1	THREAD4–WFL2	0.0007	0.0006	1.1945	−0.0017	0.0032	0.9589	0.0025	0.1417
THREAD3–WFL1	THREAD4–WFL5	0.0009	0.0008	1.2067	−0.0022	0.0041	0.9571	0.0032	0.1849
THREAD3–WFL2	THREAD3–WFL5	0.0003	0.0007	0.4346	−0.0025	0.0031	0.9996	0.0028	0.0584
THREAD3–WFL2	THREAD4–WFL1	0.0028	0.0006	4.5101	0.0003	0.0052	0.0192	0.0025	0.5370
THREAD3–WFL2	THREAD4–WFL2	0.0010	0.0005	1.8099	−0.0012	0.0032	0.7960	0.0022	0.1924
THREAD3–WFL2	THREAD4–WFL5	0.0027	0.0007	3.6073	−0.0003	0.0057	0.1125	0.0030	0.5191
THREAD3–WFL5	THREAD4–WFL1	0.0031	0.0007	4.0968	0.0000	0.0061	0.0458	0.0030	0.5954
THREAD3–WFL5	THREAD4–WFL2	0.0013	0.0007	1.8568	−0.0015	0.0041	0.7778	0.0028	0.2508
THREAD3–WFL5	THREAD4–WFL5	0.0030	0.0009	3.4728	−0.0005	0.0064	0.1404	0.0035	0.5775
THREAD4–WFL1	THREAD4–WFL2	0.0018	0.0006	2.8746	−0.0007	0.0043	0.3259	0.0025	0.3446
THREAD4–WFL1	THREAD4–WFL5	0.0001	0.0008	0.1161	−0.0031	0.0033	1.0000	0.0032	0.0179
THREAD4–WFL2	THREAD4–WFL5	0.0017	0.0007	2.2597	−0.0013	0.0047	0.6005	0.0030	0.3267

## Data Availability

The original contributions presented in this study are included in the article. Further inquiries can be directed to the corresponding author.

## References

[1] Hamrol A., Gawlik J., Sładek J. (2019). Mechanical Engineering in Industry 4.0. Manag. Prod. Eng. Rev..

[2] Takaya Y. (2013). In-Process and On-Machine Measurement of Machining Accuracy for Process and Product Quality Management: A Review. Int. J. Autom. Technol..

[3] Krawczyk B., Szablewski P., Gapiński B., Wieczorowski M., Khan R. (2024). On-Machine Measurement as a Factor Affecting the Sustainability of the Machining Process. Sustainability.

[4] Majstorovic V.D., Durakbasa N., Takaya Y., Stojadinovic S., Majstorovic V., Durakbasa N. (2019). Advanced Manufacturing Metrology in Context of Industry 4.0 Model. Proceedings of the 12th International Conference on Measurement and Quality Control—Cyber Physical Issue. IMEKOTC14 2019.

[5] Gąska A., Sładek J., Gąska P., Wang L., Majstorovic V., Mourtzis D., Carpanzano E., Moroni G., Galantucci L. (2020). Challenges for Uncertainty Determination in Dimensional Metrology Put by Industry 4.0 Revolution. Proceedings of 5th International Conference on the Industry 4.0 Model for Advanced Manufacturing.

[6] Gąska A., Sładek J. In-line and in-process metrology systems: Perspectives and challenges. Proceedings of the International Scientific—Technical Conference Manufacturing 2022.

[7] Wieczorowski M., Trojanowska J. (2023). Towards Metrology 4.0 In Dimensional Measurements. J. Mach. Eng..

[8] Horst J., Hedberg T., Feeney A.B. (2019). On-Machine Measurement Use Cases and Information for Machining Operations.

[9] Bomba G., Ornat A., Gierlak P., Muszyńska M. (2022). On-Machine Measurements for Aircraft Gearbox Machining Process Assisted by Adaptive Neuro-Fuzzy Inference System. Appl. Sci..

[10] Szyszka G., Sep J. (2022). Comparative Performance Evaluation of Multiconfiguration Touch-Trigger Probes for Closed-Loop Machining of Large Jet Engine Cases. Materials.

[11] Wieczorowski M., Eichner T., Lindner I., Pereira A. (2015). A Concept of in-Process Measurement System for Spline Forming. Manag. Prod. Eng. Rev..

[12] Kuo C.-H., Chen P.-C. (2021). On-Machine Measurement and Error Compensation for 6061 Aluminum Alloy Hexagonal Punch Using a Turn-Milling Machine. Machines.

[13] Marchewka Ł., Grudziński M. (2021). An Approach to an Intelligent Scanning of the Machine Tool Workspace. J. Mach. Eng..

[14] Krawczyk B., Smak K., Szablewski P., Gapiński B., Diering M., Wieczorowski M., Harugade M., Pereira A. (2022). Review of measurement methods to evaluate the geometry of different types of external threads. Advances in Manufacturing III.

[15] Krawczyk B., Swojak N., Gapiński B., Szablewski P., Wieczorowski M. (2026). The use of laser triangulation sensor for non-contact measurement of aerospace thread profiles under variable conditions. Measurement.

[16] Dong Z., Sun X., Chen C., Sun M. (2018). A fast and on-machine measuring system using the laser displacement sensor for the contour parameters of the drill pipe thread. Sensors.

[17] Lee Y.C., Yeh S.S. (2019). Using machine vision to develop an on-machine thread measurement system for computer numerical control lathe machines. Proceedings of the International MultiConference of Engineers and Computer Scientists.

[18] Lee Y.C., Wu Y.C., Yeh S.S. (2021). Development of an on-machine external thread measurement system for CNC lathes using eye-in-hand machine vision with morphology technology. Eng. Lett..

[19] Krawczyk B., Szablewski P., Mendak M., Gapiński B., Smak K., Legutko S., Wieczorowski M. (2023). Surface Topography Description of Threads Made with Turning on Inconel 718 Shafts. Materials.

[20] Jankowski M., Woźniak A., Mayer J.R.R. (2019). On-machine and in-laboratory investigation of errors of probes for CNC machine tools. Advances in Engineering Research and Application.

[21] Jankowski M., Woźniak A. (2016). Mechanical model of errors of probes for numerical controlled machine tools. Measurement.

[22] Jankowski M., Woźniak A., Byszewski M. (2014). Machine tool probes testing using a moving inner hemispherical master artefact. Precis. Eng..

[23] Sepahi-Boroujeni S., Mayer R., Khameneifar F., Woźniak A. (2021). A full-covariance uncertainty assessment in on-machine probing. Int. J. Mach. Tools Manuf..

[24] Jacniacka E., Semotiuk L. (2011). Odkształcenia cieplne a niedokładność pomiaru sondą przedmiotową. PAK.

[25] Jacniacka E., Semotiuk L. (2012). Powtarzalność mocowania jako składnik budżetu niepewności pomiaru sondą przedmiotową na obrabiarkach CNC. Mechanik.

[26] Jacniacka E., Semotiuk L., Babkiewicz M. (2012). Wyznaczenie dwuwymiarowej niedokładności pomiaru wewnątrzobrabiarkowego systemu pomiarowego z zastosowaniem sondy OMP60. Pomiary Autom. Robot..

[27] Holub M., Jankovych R., Andrs O., Kolibal Z. (2018). Capability Assessment of CNC Machining Centres as Measuring Devices. Measurement.

[28] Jóźwik J., Byszewski M. (2015). Badanie dokładności pozycjonowania osi obrotowych wieloosiowych obrabiarek CNC oraz błędów wolumetrycznych. Mechanik.

[29] Honczarenko J., Kwaśniewicz J. (2008). Nowe systemy pomiarowe do sprawdzania dokładności obrabiarek CNC. Mechanik.

[30] Woźniak A., Dobosz M. (2005). Influence of measured objects parameters on CMM touch trigger probe accuracy of probing. Precis. Eng..

[31] Mutilba U., Gomez-Acedo E., Kortaberria G., Olarra A., Yagüe-Fabra J.A. (2017). Traceability of On-Machine Tool Measurement: A Review. Sensors.

[32] Jankowski M., Woźniak A. (2018). Testing of the delay time of wireless communication of CNC machine tools’ probes and controller. Metrol. Meas. Syst..

[33] Woźniak A., Męczyńska K. (2020). Measurement hysteresis of touch-trigger probes for CNC machine tools. Measurement.

[34] Woźniak A., Byszewski M., Jankowski M. (2018). 3D characteristics of triggering force of CNC machine tool probe. J. Phys. Conf. Ser..

[35] Achelker B., Srinivasa Rao N., Rajendra R., Krishaniah A. (2014). Performance Evaluation of Machine Tool Probe for In-process Inspection of 2D and 3D Geometries. Procedia Technol..

[36] Liu Y., Zhao W., Sun R., Yue X. (2020). Optimal path planning for automated dimensional inspection of free-form surfaces. J. Manuf. Syst..

[37] Li L., Li B., Shi Y., Sun Z., Wei X. (2026). Compensation and measurement of external cylindrical thread geometric errors based on machine vision and optical micrometer. Measurement.

[38] Wang X., Wang C., Luo W., Sang P., Long H., Huang J., Shu J., He X. An Efficient Method for Measuring Oil Casing Thread Geometric Parameters Using Point Cloud Data. Proceedings of the 2025 10th International Conference on Image, Vision and Computing (ICIVC).

[39] Li X., Zhou J., Li W., Ma X., Yuan X., Yin X., Chen H., Chen X., Yang Y. (2025). A full-morphology measurement method and system for tubing internal thread based on rotating-mirrored structured light vision. Measurement.

[40] Li L., Liu Z., Qian X. (2024). Online Inspection Method and System Design for Screw Threads of Rebar Head Based on Machine Vision. Buildings.

[41] Renishaw. https://store.renishaw.com/en-FR/product/A-5000-8663.

[42] (2017). Buttress Inch Screw Threads.

[43] Krawczyk B. (2025). Metodyka Pomiaru Gwintów Zewnętrznych na Tokarkach CNC. Ph.D. Thesis.

